# Differentiation of AB-FUBINACA and its five positional isomers using liquid chromatography–electrospray ionization-linear ion trap mass spectrometry and triple quadrupole mass spectrometry

**DOI:** 10.1007/s11419-018-0410-4

**Published:** 2018-03-02

**Authors:** Takaya Murakami, Yoshiaki Iwamuro, Reiko Ishimaru, Satoshi Chinaka, Nariaki Takayama, Hiroshi Hasegawa

**Affiliations:** 1 Forensic Science Laboratory, Ishikawa Prefectural Police H.Q., 1-1 Kuratsuki, Kanazawa, Ishikawa 920-8553 Japan; 2 Institute of Science and Engineering, Kanazawa University, Kakuma, Kanazawa, Ishikawa 920-1192 Japan

**Keywords:** AB-FUBINACA, Positional isomer differentiation, Liquid chromatography, Electrospray ionization, Linear ion trap mass spectrometry, Triple quadrupole mass spectrometry

## Abstract

**Purpose:**

Positional isomer differentiation is crucial for forensic analysis. The aim of this study was to differentiate AB-FUBINACA positional isomers using liquid chromatography (LC)–electrospray ionization (ESI)-linear ion trap mass spectrometry (LIT-MS) and LC–ESI-triple quadrupole mass spectrometry (QqQ-MS).

**Methods:**

AB-FUBINACA, its two fluorine positional isomers on the phenyl ring, and three methyl positional isomers in the carboxamide side chain were analyzed by LC–ESI-LIT-MS and LC–ESI-QqQ-MS.

**Results:**

Four of the positional isomers, excluding AB-FUBINACA and its 3-fluorobenzyl isomer, were chromatographically separated on an ODS column in isocratic mode. ESI-LIT-MS could discriminate only three isomers, i.e., the 2-fluorobenzyl isomer, the *N*-(1-amino-2-methyl-1-oxobutan-2-yl) isomer, and the *N*-(1-amino-1-oxobutan-2-yl)-*N*-methyl isomer, based on their characteristic product ions observed at the MS^3^ stage in negative mode. ESI-QqQ-MS differentiated all six isomers in terms of the relative abundances of the product ions that contained the isomeric moieties involved in collision-induced dissociation reactions. The six isomers were more clearly and significantly differentiated upon comparison of the logarithmic values of the product ion abundance ratios as a function of collision energy.

**Conclusions:**

The present LC–MS methodologies were useful for the differentiation of a series of AB-FUBINACA positional isomers.

**Electronic supplementary material:**

The online version of this article (10.1007/s11419-018-0410-4) contains supplementary material, which is available to authorized users.

## Introduction

Over the past 50 years, pharmaceutical companies and academic laboratories have developed synthetic cannabinoids (SCs) as potential pharmaceutical agents for the treatment of pain. These compounds are CB_1_ and/or CB_2_ agonists and elicit similar effects to that of Δ^9^-tetrahydrocannabinol (THC), the active component in cannabis [1–3]. In late 2008, the European Monitoring Center for Drugs and Drug Addiction (EMCDDA) detected several SCs, including JWH-018, JWH-073, and CP-47,497, in herbal smoking mixtures marketed as incense or room odorizers often known as ‘K2’ or ‘Spice’. Subsequently, various herbal products containing SCs have been distributed worldwide [4], resulting in numerous serious incidents involving individuals under their influence. To immediately deter the epidemic of drug abuse, many countries have increased the number of individually named controlled substances. However, structurally distinct designer drugs aimed at circumventing such legal measures have continued to be synthesized. In an attempting to address worldwide social concerns, the UK government has implemented a blanket scheduling of SCs through the introduction of generic legislation based on modifications to core structures, as recommended by the UK home office from the Advisory Council on the Misuse of Drugs (ACMD) in 2009 [5]. However, this regulation did not deter the prevalence of SCs. Indeed, according to reports from the Forensic Early Warning System (FEWS) in 2012, noncontrolled SCs such as AM2201, RCS-4, and UR-144, so-called ‘second-generation’ synthetic cannabinoids, were frequently detected in forensic seizures [6–8].

**Fig. 1:**
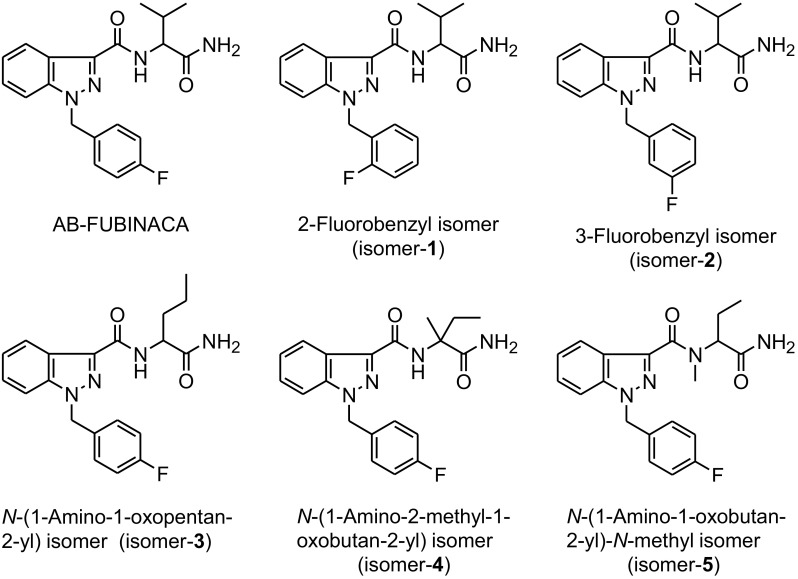
Structures of AB-FUBINACA and its five positional isomers

A new designer indazole-type SC drug bearing a 4-fluorobenzyl group at the *N*-1 position, *N*-(1-amino-3-methyl-1-oxobutan-2-yl)-1-(4-fluorobenzyl)-1*H*-indazole-3- carboxamide (AB-FUBINACA), which circumvents such blanket laws, was first identified in Japan from herbal products [9]. It was developed and patented by Pfizer Inc. as an agonist to the CB_1_ receptor, which has a much higher affinity for this receptor (*K*_i_ = 0.9 nM) than THC (*K*_i_ = 41 nM) [10]. AB-FUBINACA was one of the most identified components of herbal products in early 2012, and was subsequently scheduled as a ‘designated substance’ under the Pharmaceutical and Medical Device Act (previously the Pharmaceutical Affairs Law) in December 2012 in Japan. Its 2-fluorobenzyl isomer was also identified domestically and abroad according to the Japanese Ministry of Health, Labor and Welfare, and was listed by the law as a designated substance in December 2015. Clearly, the law requires the accurate identification of AB-FUBINACA isomers. In this regard, nuclear magnetic resonance spectroscopy is often used to determine the structures of analytes, but it is not suitable for forensic samples because of their minute amounts or high levels of impurities. Additionally, AB-FUBINACA isomers are difficult to differentiate, because they exhibit similar retention properties and mass spectral patterns [11]. To overcome these difficulties, we focused on the functions of linear ion trap mass spectrometry (LIT-MS) and triple quadrupole mass spectrometry (QqQ-MS). LIT-MS can be used for multiple-stage mass spectrometry (MS^n^) simply by using additional operations performed sequentially. This method varies the ion/molecule reaction time to delineate the kinetics, equilibrium, and fragmentation mechanisms [12]. On the other hand, QqQ-MS provides good control over the kinetic energies of the ions by controlling the given collision energy (CE) [12]. Thus far, several research groups have differentiated positional isomers of other abused drugs using LIT-MS and/or QqQ-MS combined with gas chromatography (GC) or liquid chromatography (LC) [13–16]. Previously, we developed the method of distinguishing the 2-, 3-, and 4-fluorine substitution positions on the phenyl ring in SCs containing a fluorobenzyl group, such as AB-FUBINACA and its 2- and 3-fluorobenzyl isomers, based on the so-called energy-resolved mass spectrometry (ERMS) strategy [17–21] using GC–electron ionization (EI)-QqQ-MS [22, 23]. We speculated that the methodology should be used in combination with other chromatographic and ionization techniques. Therefore, in this study, we employed LC–electrospray ionization (ESI)-LIT-MS and LC–ESI-QqQ-MS, and investigated their viability in the differentiation of AB-FUBINACA positional isomers, including methyl positional isomers in the carboxamide side chain, as well as fluorine positional isomers on the phenyl ring (Fig. 1). This is the first report on the differentiation of the positional isomers of an indazole-type SC containing a fluorobenzyl group at *N*-1 position using LC–MS systems.

## Materials and methods

### Reagents

*N*-(1-Amino-3-methyl-1-oxobutan-2-yl)-1-(4-fluorobenzyl)-1*H*-indazole-3-carboxamide (AB-FUBINACA), *N*-(1-amino-3-methyl-1-oxobutan-2-yl)-1-(2-fluorobenzyl)-1*H*-indazole-3-carboxamide (isomer-**1**), *N*-(1-amino-3-methyl-1-oxobutan-2-yl)-1-(3-fluorobenzyl)-1*H*-indazole-3-carboxamide (isomer-**2**), *N*-(1-amino-1-oxopentan-2-yl)-1-(4-fluorobenzyl)-1*H*-indazole-3-carboxamide (isomer-**3**), *N*-(1-amino-2-methyl-1-oxobutan-2-yl)-1-(4-fluorobenzyl)-1*H*-indazole-3-carboxamide (isomer-**4**), and *N*-(1-amino-1-oxobutan-2-yl)-1-(4-fluorobenzyl)-*N*-methyl-1*H*-indazole-3-carboxamide (isomer-**5**) (Fig. 1) were purchased from Cayman Chemical (Ann Arbor, MI, USA). Their standard stock solutions (200 μg/mL) were prepared in methanol and stored at − 20 °C. The working standard solutions (20 μg/mL) to be injected into the mass spectrometer were prepared by diluting the stock solutions.

### LC–ESI-LIT-MS

LC–ESI-LIT-MS was performed on a Prominence Ultrafast Liquid Chromatograph (Shimadzu, Kyoto, Japan) linked to an LXQ LIT mass spectrometer (Thermo Fisher Scientific, Waltham, MA, USA) equipped with an ESI source. Instrumental control, data acquisition, and analysis were performed using Xcalibur software ver. 2.0 (Thermo Fisher Scientific). The analytes were separated using two ODS columns: (1) L-column 2 ODS column (150 × 1.5 mm i.d., particle size 5 μm; Chemicals Evaluation and Research Institute, Tokyo, Japan) and (2) YMC-Ultra HT Pro C18 column (75 × 2.0 mm i.d., particle size 2 μm; YMC, Kyoto, Japan) at a column oven temperature of 40 °C. The injection volume was 1 μL. Other LC parameters using the (1) L-column 2 ODS column and (2) YMC-Ultra HT Pro C18 column were as follows, respectively: (1) flow rate, 0.1 mL/min; elution mode, gradient with 10 mM ammonium acetate/5% methanol in distilled water (A) and 10 mM ammonium acetate/5% distilled water in methanol (B) from 100% A to 100% B over 15 min, and by isocratic elution with the final solvent composition for 10 min; and (2) flow rate, 0.25 mL/min; elution mode, isocratic with 50% A/50% B for 30 min. The MS parameters were as follows: polarity, positive and negative; scan mode, product ion scan; activation type, collision-induced dissociation (CID); isolation width, *m*/*z* 2.00; normalized CE, 35.0%; activation Q, 0.250; activation time, 30 ms; collision gas, helium.

### LC–ESI-QqQ-MS

LC–ESI-QqQ-MS was performed on an Agilent 1260 Infinity LC system linked to a 6470A triple quadrupole LC/MS tandem mass spectrometer (Agilent Technologies, Santa Clara, CA, USA) equipped with an ESI source. Instrumental control, data acquisition, and analysis were performed using Mass Hunter software ver. B.07.00 (Agilent Technologies). An L-column 2 ODS column (150 × 1.5 mm i.d., particle size 5 μm; Chemicals Evaluation and Research Institute) was used at a column oven temperature of 40 °C. The injection volume was 5 μL. The flow rate was 0.1 mL/min. The elution mode was isocratic with 10 mM ammonium acetate/60% methanol in distilled water for 25 min. The MS parameters were as follows: polarity, positive; scan mode, product ion scan; fragmentor voltage, 80 eV; cell accelerator voltage, 5 eV; collision gas, nitrogen; CE, 0–90 eV.

### Statistical calculations

Statistical analyses were performed using BellCurve for Excel ver. 2.02 (Social Survey Research Information Co., Ltd., Tokyo, Japan), which is an add-in software to Microsoft Excel 2010. The homogeneity of variances was calculated by Bartlett’s test to determine if the obtained data were normally distributed (parametric, *p* > 0.05) or not (nonparametric, *p* < 0.05). If the data were parametric, one-way analysis of variance (ANOVA) followed by multiple pairwise comparisons as post hoc analysis using Tukey’s test was performed. For nonparametric data, Kruskal–Wallis test as nonparametric ANOVA, followed by the Steel–Dwass test as a nonparametric multiple pairwise comparison, was performed.

## Results and discussion

### Liquid chromatography

**Fig. 2:**
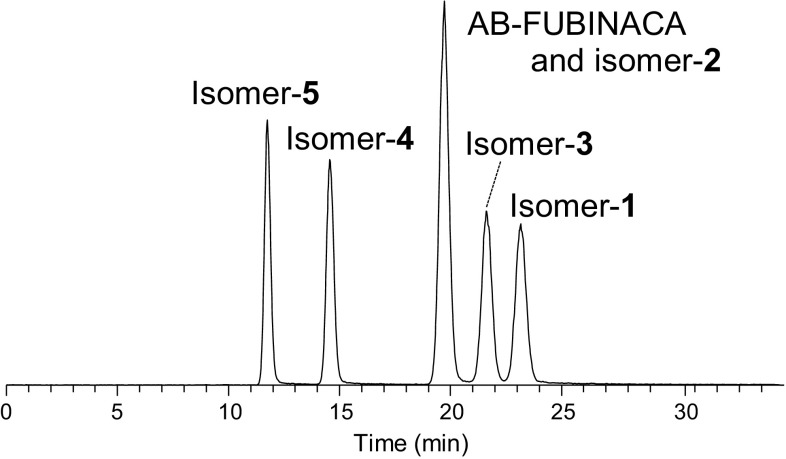
Extracted ion chromatogram ([M+H]^+^; *m*/*z* 369) of a mixture of AB-FUBINACA and its five positional isomers using a YMC-Ultra HT Pro C18 column in isocratic mode

For drug screening analysis, we typically use a L-column 2 ODS column in gradient mode with 10 mM ammonium acetate/5% methanol in distilled water (A) and 10 mM ammonium acetate/5% distilled water in methanol (B) from 100% A to 100% B over 15 min, followed by isocratic elution with the final solvent composition for 10 min. The extracted ion chromatogram ([M+H]^+^; *m*/*z* 369) of a mixture of the six AB-FUBINACA isomers is shown in Fig. S1. The six isomers coeluted and could not be baseline separated. Although isomer-**4** and isomer-**5** overlapped at the peak shoulders, they were distinguished at the peak apexes. The existence of isomer-**1** was recognized, but the peak was buried in the group of peaks for AB-FUBINACA, isomer-**2**, and isomer-**3**. Among other ODS columns and mobile-phase compositions, a YMC-Ultra HT Pro C18 column operated in isocratic mode with 50% A/50% B achieved chromatographic separation among isomers-**1**, -**3**, -**4**, and -**5**, while AB-FUBINACA and isomer-**2** still coeluted (Fig. 2). Their elution order was isomer-**5** < isomer-**4** < AB-FUBINACA and isomer-**2** < isomer-**3** < isomer-**1**. The coelution of AB-FUBINACA and isomer-**2** resulted from the minimal interaction strength difference with the ODS column surface, because of their high conformational similarity.

### Linear ion trap multiple-stage mass spectrometry

ESI-LIT mass spectra of the six isomers were recorded in positive mode. The precursor ions at the MS^2^–MS^6^ stages were set at *m*/*z* 369, 352, 324, 253, and 225, respectively. The obtained mass spectra in the MS^1^–MS^6^ stages are shown in Fig. S2. Spectra in stages greater than MS^7^ were not obtained because of the lack of product ions. In the mass spectra of each isomer, identical fragment/product ions were observed at *m*/*z* 369 ([M+H]^+^) and 391 ([M+Na]^+^) in the MS^1^ stage, at *m*/*z* 352 ([M–NH_2_]^+^) in MS^2^, at *m*/*z* 324 ([M–CONH_2_]^+^) in MS^3^, at *m*/*z* 253 ([M–(C_4_H_9_N)CONH_2_]^+^) in MS^4^, at *m*/*z* 109 (fluorobenzyl cation), 225 ([M–CO(C_4_H_9_N)CONH_2_]^+^) and 235 in MS^5^, and at *m*/*z* 198 and 205 in MS^6^. Although the abundances of the ions at *m*/*z* 198 in MS^6^ appeared to slightly differ in isomer-**2**, the other mass spectra were very similar. Therefore, ESI-LIT-MS operated in positive mode could not be used to effectively differentiate the six isomers.

**Fig. 3:**
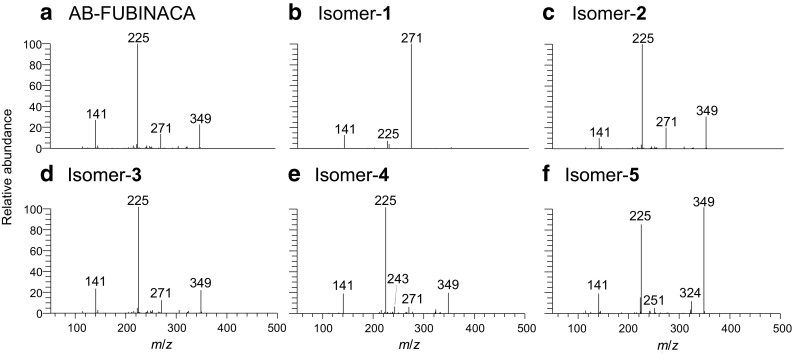
Electrospray ionization-linear ion trap MS^3^ spectra of AB-FUBINACA and its five positional isomers in negative mode. The precursor ion was set at *m*/*z* 367

Mass spectra of the six isomers were also obtained in negative mode in the MS^1^–MS^3^ stages (Fig. S3). Note that spectra at stages greater than MS^4^ were not consistently available, because of the lack of product ions. The precursor ions at MS^2^ and MS^3^ stages were set at *m*/*z* 427 and 367, respectively. The acetate adduct ions at *m*/*z* 427 ([M+CH_3_COO]^−^) at MS^1^ and the deprotonated ions at *m*/*z* 367 ([M–H]^−^) at MS^2^ were observed as base peaks for all the isomers. At the MS^3^ stage, some spectral differences among isomers-**1**, -**4**, and -**5** were observed (Fig. 3). Figure 3b shows that the *m*/*z* 271 ion of isomer-**1** was detected as the base peak, whereas the *m*/*z* 349 ion was hardly detected. Furthermore, the abundances of the *m*/*z* 141 and 225 ions were lower than those of the other isomers. Isomer-**4** showed a characteristic ion at *m*/*z* 243 (Fig. 3e). Isomer-**5** showed characteristic ions at *m*/*z* 251 and 324, whereas the *m*/*z* 271 ion was barely detected. In addition, the relative abundance of the *m*/*z* 349 ion was higher than that of AB-FUBINACA, isomer-**2**, -**3**, or -**4**, which constituted the base peak (Fig. 3f). It was difficult to differentiate among AB-FUBINACA, isomer-**2**, and isomer-**3** because of their similar spectral patterns, although the relative abundance of the *m*/*z* 141 ion of isomer-**2** was slightly lower (Fig. 3a, c, d).

### Triple quadrupole energy-resolved mass spectrometry

**Fig. 4:**
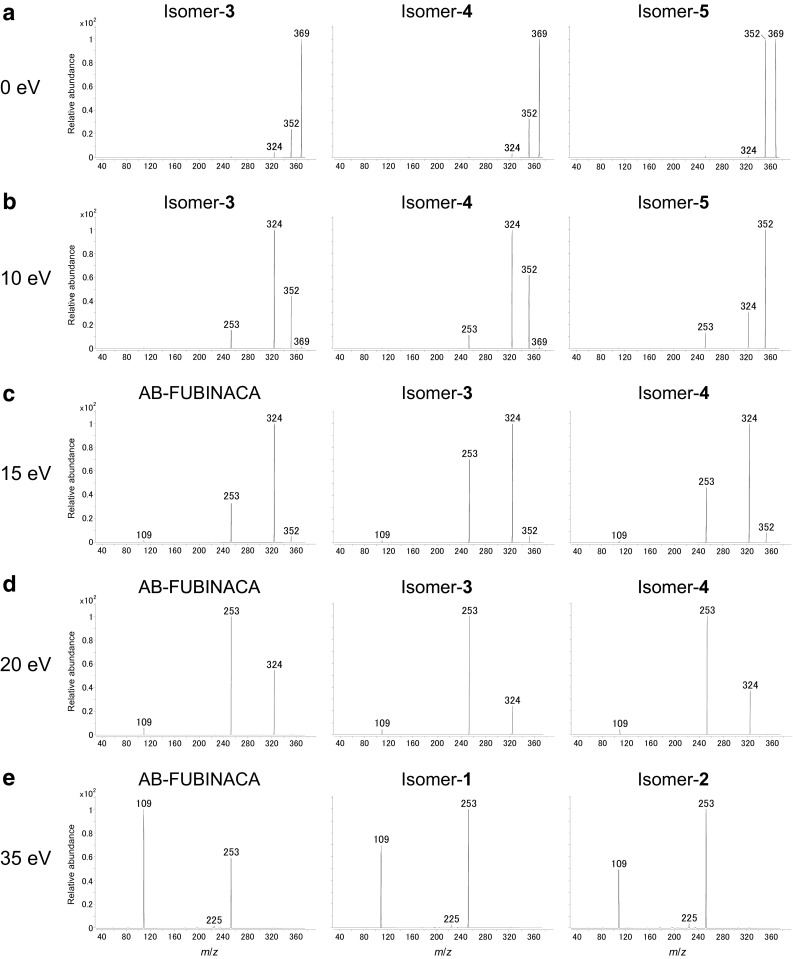
Electrospray ionization-triple quadrupole mass spectra of **a** isomers-**3**, -**4**, and -**5** at a collision energy of 0 eV, **b** isomers-**3**, -**4**, and -**5** at 10 eV, **c** AB-FUBINACA, isomer-**3**, and isomer-**4** at 15 eV, **d** AB-FUBINACA, isomer-**3**, and isomer-**4** at 20 eV, and **e** AB-FUBINACA, isomer-**1**, and isomer-**2** at 35 eV for the precursor ion at *m*/*z* 369 in positive mode

The ERMS strategy makes it possible to reveal the behaviors of product ions. The product ion spectra for the precursor ions at *m*/*z* 369 were measured with the CE setting at 0–90 eV (0–40 eV in increments by every 5 eV, and 50, 70, and 90 eV). All the obtained spectra are shown in Fig. S4, and several representative mass spectra are shown in Fig. 4. As a reference, the breakdown curves are also shown in Fig. S5. The observed product ions for the six isomers at all CEs were identical. However, careful comparison of the relative abundances of the product ions enabled us to determine the dissociation characteristics of each isomer. As seen in the spectra at 0 and 5 eV, the relative abundances of the two ions at *m*/*z* 352 and 369 of isomer-**5** were clearly different from the other isomers (Fig. 4a, S4). This indicates that it was easier to eliminate the terminal amine in the carboxamide side chain of the precursor ion at *m*/*z* 369 of isomer-**5**, because the dissociation energy from *m*/*z* 369 to 352 was lower than the other isomers due to methylation of the amine in the amido bond. Among the four isomers except isomer-**5**, the relative abundance of the *m*/*z* 352 ion for isomer-**4** was the highest at a CE of 0 eV and those of the *m*/*z* 324 and 369 ions were lowest at 5 eV. AB-FUBINACA, and isomers-**1**, -**2**, and -**3** exhibited minimal differences in their spectra at 0 and 5 eV. The relative abundance of the *m*/*z* 324 ion of isomer-**5** at 10 eV was much lower than that of the other isomers (Fig. 4b, S4), indicating that isomer-**5** required the highest dissociation energy to eliminate the carbonyl group from the *m*/*z* 352 ion. Comparing isomer-**3** with isomer-**4**, the relative abundance of the *m*/*z* 352 ion of isomer-**3** was lower at 10 eV, which suggested that the tertiary carbon–carbonyl carbon bond was easier to cleave than the quaternary carbon–carbonyl carbon bond. At a CE of 15 eV, the *m*/*z* 253 ion of the isomer-**5** showed the highest abundance among the six isomers (Fig. S4), suggesting that the *m*/*z* 253 ion was generated easily upon cleavage of the *m*/*z* 324 ion. Except for isomer-**5**, the relative abundances of the *m*/*z* 253 ions at 15 eV were in the order of AB-FUBINACA < isomer-**4** < isomer-**3** (Fig. 4c), whereas those of the *m*/*z* 324 ions at 20 eV were isomer-**3** < isomer-**4** < AB-FUBINACA (Fig. 4d). Additionally, the relative abundances of the ions at *m*/*z* 109 and 253 varied among the three isomers at 20–50 eV. The abundances of the ions at *m*/*z* 109 and 253 were in the order of isomer-**2** < isomer-**1** < AB-FUBINACA at 20–35 eV (Fig. 4e, S4) and AB-FUBINACA < isomer-**1** < isomer-**2** at 40 and 50 eV (Fig. S4), respectively. This ordering was due to the differences in energy required to cleave the indazole moiety from the fluorobenzyl group, which we attribute to a halogen electron-donating resonance effect [22].

**Fig. 5:**
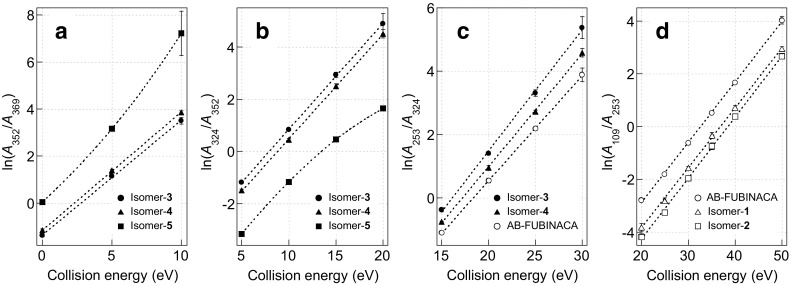
Plots of the logarithmic values of the abundance ratios of **a** *m*/*z* 352 to 369 at collision energies of 0–10 eV for isomer-**3**, -**4**, and -**5**, **b** *m*/*z* 324 to 352 at 5–20 eV for isomer-**3**, -**4**, and -**5**, **c** *m*/*z* 253 to 324 at 15–30 eV for AB-FUBINACA, isomer-**3**, and isomer-**4**, and **d** *m*/*z* 109 to 253 at 20–50 eV for AB-FUBINACA, isomer-**1**, and isomer-**2**. Error bars represent twice the standard errors (*n* = 5)

In order to further clarify the abovementioned relationships, the natural logarithmic values of the abundance ratios of the product ions involved in CID reactions, assuming that precursor ions were quantitatively cleaved in accordance with Gibbs free energy equation, were plotted as a function of CE. First, the logarithmic values of the abundance ratios of the ion at *m*/*z* 352 to 369 [*ln*(*A*_352_/*A*_369_)] in the CE range of 0–10 eV for isomers-**3**, -**4**, and -**5** revealed the relation of isomer-**3** < isomer-**4** < isomer-**5** (Fig. 5a). To demonstrate significant differences among the *ln*(*A*_352_/*A*_369_) values of the three isomers, the homogeneity of variance test, analysis of variance (ANOVA), and multiple pairwise comparisons were performed. Bartlet’s test for homogeneity of variances showed that the *ln*(*A*_352_/*A*_369_) values at CEs of 0 and 5 eV were normally distributed (parametric) and the values at 10 eV were nonparametric. For the parametric data at 0 and 5 eV, one-way ANOVA showed significant differences in the *ln*(*A*_352_/*A*_369_) values among the three isomers (significance *α* < 2.4 × 10^−17^, Table S1), and Tukey’s test revealed low *p* values (*p* < 1.7 × 10^−6^, Table S1). For the nonparametric data at 10 eV, the Kruskal–Wallis test followed by the Steel–Dwass test also showed that the three isomers were significantly different (*α* = 1.9 × 10^−3^, *p* < 2.5 × 10^−2^, Table S1). Second, the logarithmic values of the product ion abundance ratios of *m*/*z* 324 to 352 [*ln*(*A*_324_/*A*_352_)] for isomers-**3**, -**4**, and -**5** were plotted in the CE range of 5–20 eV. The *ln*(*A*_324_/*A*_352_) plots were also significantly separated in the order of isomer-**5** < isomer-**4** < isomer-**3** (Fig. 5b, Table S2). Third, the logarithmic values of the abundance ratios of *m*/*z* 253 to 324 [*ln*(*A*_253_/*A*_324_)] in 15–30 eV for AB-FUBINACA, isomer-**3**, and isomer-**4** were plotted and showed significant differences in the order of AB-FUBINACA < isomer-**4** < isomer-**3** (Fig. 5c, Table S3). Lastly, the logarithmic values of the abundance ratios of *m*/*z* 109 to 253 [*ln*(*A*_109_/*A*_253_)] for AB-FUBINACA, isomer-**1**, and isomer-**2** showed a significant relationship of isomer-**2** < isomer-**1** < AB-FUBINACA in the CE range of 20–50 eV (Fig. 5d, Table S4), which agrees with our previous analyses using GC–EI-QqQ-MS, although the internal energies of the precursor ions differed on account of the difference of ionization techniques employed [22, 23]. The logarithmic values of the abundance ratios for the five isomers, except isomer-**5**, revealed a linear relationship with the CE with high correlation coefficients (Fig. 5). The reliabilities of the data were ensured by good repeatability of the mass spectral data. The relative standard deviations (%RSD) of the abundances of the product ions were low (Tables S5–S8), although some data such as *m*/*z* 369 at 10 eV for isomer-**5** in Table S5, *m*/*z* 352 at 20 eV for isomer-**3** in Table S6, and *m*/*z* 324 at 30 eV for isomer-**3** in Table S7 were comparatively dispersed, because of their low abundance. Twice the standard deviations of the logarithmic values were also very low, as shown in Tables S5–S8. Therefore, comparing the logarithmic values at each range of CEs enabled the six isomers to be differentiated clearly and reliably.

## Conclusions

We have investigated the differences between AB-FUBINACA and its five positional isomers (two fluorine positional isomers on the phenyl ring and three methyl positional isomers in the carboxamide side chain) by LC–ESI-LIT-MS and LC–ESI-QqQ-MS. Excluding AB-FUBINACA and its 3-fluorobenzyl isomer (isomer-**2**), four of the isomers were separated on an ODS column operated in isocratic mode. Multiple-stage mass spectrometry using LIT in negative mode allowed three isomers, namely, the 2-fluorobenzyl isomer (isomer-**1**), the *N*-(1-amino-2-methyl-1-oxobutan-2-yl) isomer (isomer-**4**), and the *N*-(1-amino-1-oxobutan-2-yl)-*N*-methyl isomer (isomer-**5**), to be differentiated based on their characteristic product ions observed in the MS^3^ stage. Energy-resolved mass spectrometry, which employs QqQ-MS as a function of CE, revealed that the relative abundances of the product ions containing the isomeric moieties, produced by CID reactions, were different for the six isomers. Furthermore, all six isomers were clearly differentiated by plotting the logarithmic values of the characteristic product ion abundance ratios against the CE. Based on the above results, the combination of LC with ESI-QqQ-MS is effective for the differentiation of a series of AB-FUBINACA positional isomers.

## Electronic supplementary material

Below is the link to the electronic supplementary material.

Supplementary material 1 (PDF 635 kb)
