# Supplementary material for: Differentiation of AB-FUBINACA and its five positional isomers using liquid chromatography–electrospray ionization-linear ion trap mass spectrometry and triple quadrupole mass spectrometry
Source: Forensic Toxicol. 2018 Mar 2;36(2):351–8. doi: 10.1007/s11419-018-0410-4 (PMC6002426; doi:10.1007/s11419-018-0410-4)
Supplement: Supplementary file 1 — Supplementary material 1 (PDF 635 kb) [file 11419_2018_410_MOESM1_ESM.pdf]

Electronic supplementary material

Differentiation of AB-FUBINACA and its five positional isomers using liquid chromatography–electrospray ionization-linear ion trap mass spectrometry and triple quadrupole mass spectrometry

Takaya Murakami[1,2], Yoshiaki Iwamuro[1], Reiko Ishimaru[1], Satoshi Chinaka[1], Nariaki Takayama[1], and Hiroshi Hasegawa[2]

[1] Forensic Science Laboratory, Ishikawa Prefectural Police H.Q., 1-1 Kuratsuki, Kanazawa, Ishikawa 920-8553, Japan
[2] Institute of Science and Engineering, Kanazawa University, Kakuma, Kanazawa, Ishikawa 920-1192, Japan

E-mail: murakami@forensic-sci-lab.net (T. Murakami)

Table S1 Results of ANOVA and multiple pairwise comparisons of the logarithmic values of the abundance ratios of *m*/*z* 352 to 369 for isomers-**3**, -**4**, and -**5**.

Collision energy (eV)	ANOVA[a] (α)	Multiple pairwise comparisons[b] (p)		
		Isomer-**3** × -**4**	Isomer-**3** × -**5**	Isomer-**4** × -**5**
0	2.4×10^{-17}	1.8×10^{-6}	1.7×10^{-6}	1.7×10^{-6}
5	2.0×10^{-18}	2.0×10^{-6}	1.7×10^{-6}	1.7×10^{-6}
10	1.9×10^{-3}	2.5×10^{-2}	2.5×10^{-2}	2.5×10^{-2}

[a] one-way ANOVA (0 and 5 eV), Kruskal–Wallis test (10 eV)

[b] Tukey's test (0 and 5 eV), Steel–Dwass test (10 eV)

Table S2 Results of ANOVA and multiple pairwise comparisons of the logarithmic values of the abundance ratios of *m*/*z* 324 to 352 for isomers-**3**, -**4**, and -**5**.

Collision energy (eV)	ANOVA[a] (α)	Multiple pairwise comparisons[b] (p)		
		Isomer-**3** × -**4**	Isomer-**3** × -**5**	Isomer-**4** × -**5**
5	1.7×10^{-18}	1.7×10^{-6}	1.7×10^{-6}	1.7×10^{-6}
10	2.9×10^{-20}	1.7×10^{-6}	1.7×10^{-6}	1.7×10^{-6}
15	1.5×10^{-17}	1.7×10^{-6}	1.7×10^{-6}	1.7×10^{-6}
20	1.9×10^{-3}	2.5×10^{-2}	2.5×10^{-2}	2.5×10^{-2}

[a] one-way ANOVA (5, 10, and 15 eV), Kruskal–Wallis test (20 eV)

[b] Tukey's test (5, 10, and 15 eV), Steel–Dwass test (20 eV)

Table S3 Results of ANOVA and multiple pairwise comparisons of the logarithmic values of the abundance ratios of *m*/*z* 253 to 324 for AB-FUBINACA, isomer-**3**, and isomer-**4**.

Collision energy (eV)	ANOVA[a] (α)	Multiple pairwise comparisons[b] (p)		
		AB-FUBINACA × isomer-**3**	AB-FUBINACA × isomer-**4**	Isomer-**3** × -**4**
15	1.2×10^{-14}	1.7×10^{-6}	1.7×10^{-6}	1.7×10^{-6}
20	1.2×10^{-13}	1.7×10^{-6}	1.7×10^{-6}	1.7×10^{-6}
25	8.1×10^{-15}	1.7×10^{-6}	1.7×10^{-6}	1.7×10^{-6}
30	1.1×10^{-9}	1.7×10^{-6}	5.1×10^{-6}	2.6×10^{-6}

[a] one-way ANOVA

[b] Tukey's test

Table S4 Results of ANOVA and multiple pairwise comparisons of the logarithmic values of the abundance ratios of *m/z* 109 to 253 for AB-FUBINACA, isomer-**1**, and isomer-**2**.

Collision energy (eV)	ANOVA[a] (α)	Multiple pairwise comparisons[b] (p)		
		AB-FUBINACA × isomer-**1**	AB-FUBINACA × isomer-**2**	Isomer-**1** × -**2**
20	9.6×10^{-14}	1.7×10^{-6}	1.7×10^{-6}	2.6×10^{-6}
25	4.5×10^{-16}	1.7×10^{-6}	1.7×10^{-6}	1.7×10^{-6}
30	4.2×10^{-15}	1.7×10^{-6}	1.7×10^{-6}	1.7×10^{-6}
35	7.1×10^{-14}	1.7×10^{-6}	1.7×10^{-6}	1.8×10^{-6}
40	2.9×10^{-16}	1.7×10^{-6}	1.7×10^{-6}	1.7×10^{-6}
50	2.3×10^{-14}	1.7×10^{-6}	1.7×10^{-6}	8.6×10^{-6}

[a] one-way ANOVA

[b] Tukey's test

Table S5 Relative standard deviations (%RSD) of the abundances of the *m/z* 352 and 369 ions and the logarithmic values of the abundance ratios ($ln(A_{352}/A_{369})$) with twice the standard deviation (SD) at collision energies of 0, 5, and 10 eV for isomers-**3**, -**4**, and -**5**.

Collision energy (eV)	Isomer-**3**			Isomer-**4**			Isomer-**5**		
	%RSD		$ln(A_{352}/A_{369})$ ± 2SD	%RSD		$ln(A_{352}/A_{369})$ ± 2SD	%RSD		$ln(A_{352}/A_{369})$ ± 2SD
	m/z 352	*m/z* 369		*m/z* 352	*m/z* 369		*m/z* 352	*m/z* 369	
0	2.2	2.6	-1.38 ± 0.06	3.4	1.9	-1.15 ± 0.06	2.2	3.4	0.04 ± 0.05
5	2.2	3.0	1.15 ± 0.04	1.1	1.9	1.38 ± 0.06	1.5	4.9	3.15 ± 0.09
10	2.8	8.6	3.50 ± 0.14	2.4	3.6	3.86 ± 0.06	2.5	47	7.21 ± 0.95

Table S6 %RSD of the abundances of the *m/z* 324 and 352 ions and the logarithmic values of the abundance ratios ($ln(A_{324}/A_{352})$) with twice the SD at collision energies of 5–20 eV for isomers-**3**, -**4**, and -**5**.

Collision energy (eV)	Isomer-**3**			Isomer-**4**			Isomer-**5**		
	%RSD		$ln(A_{324}/A_{352})$ ± 2SD	%RSD		$ln(A_{324}/A_{352})$ ± 2SD	%RSD		$ln(A_{324}/A_{352})$ ± 2SD
	m/z 324	*m/z* 352		*m/z* 324	*m/z* 352		*m/z* 324	*m/z* 352	
5	2.1	2.2	-1.19 ± 0.03	3.4	1.1	-1.50 ± 0.05	5.0	1.5	-3.16 ± 0.09
10	2.8	2.8	0.83 ± 0.03	2.6	2.4	0.45 ± 0.04	1.6	2.5	-1.17 ± 0.06
15	1.6	6.1	2.93 ± 0.10	2.9	2.4	2.50 ± 0.09	3.1	3.7	0.46 ± 0.09
20	3.0	20	4.96 ± 0.39	1.5	8.1	4.50 ± 0.16	2.8	4.4	1.65 ± 0.08

Table S7 %RSD of the abundances of the *m/z* 253 and 324 ions and the logarithmic values of the abundance ratios ($ln(A_{253}/A_{324})$) with twice the SD at collision energies of 15–30 eV for AB-FUBINACA, isomer-**3**, and isomer-**4**.

Collision energy (eV)	AB-FUBINACA			Isomer-**3**			Isomer-**4**		
	%RSD		$ln(A_{253}/A_{324})$ ± 2SD	%RSD		$ln(A_{253}/A_{324})$ ± 2SD	%RSD		$ln(A_{253}/A_{324})$ ± 2SD
	m/z 253	*m/z* 324		*m/z* 253	*m/z* 324		*m/z* 253	*m/z* 324	
15	1.8	2.6	-1.11 ± 0.04	3.9	1.6	-0.38 ± 0.06	3.6	2.8	-0.76 ± 0.03
20	1.8	2.8	0.54 ± 0.07	2.1	3.0	1.40 ± 0.05	4.2	1.5	0.95 ± 0.08
25	1.6	1.1	2.18 ± 0.05	3.9	4.5	3.31 ± 0.10	2.1	0.9	2.72 ± 0.04
30	2.9	8.0	3.89 ± 0.21	2.4	16	5.37 ± 0.35	0.9	6.5	4.58 ± 0.13

Table S8 %RSD of the abundances of the *m/z* 109 and 253 ions and the logarithmic values of the abundance ratios ($ln(A_{109}/A_{253})$) with twice the SD at collision energies of 20–50 eV for AB-FUBINACA, isomer-**1**, and isomer-**2**.

Collision energy (eV)	AB-FUBINACA			Isomer-**1**			Isomer-**2**		
	%RSD		$ln(A_{109}/A_{253})$ ± 2SD	%RSD		$ln(A_{109}/A_{253})$ ± 2SD	%RSD		$ln(A_{109}/A_{253})$ ± 2SD
	m/z 109	*m/z* 253		*m/z* 109	*m/z* 253		*m/z* 109	*m/z* 253	
20	4.1	1.8	-2.78 ± 0.06	6.6	3.0	-3.82 ± 0.14	8.0	2.6	-4.17 ± 0.11
25	2.1	1.6	-1.81 ± 0.03	4.4	1.7	-2.79 ± 0.09	2.5	3.3	-3.26 ± 0.08
30	2.0	2.9	-0.63 ± 0.07	2.4	1.6	-1.55 ± 0.06	4.6	2.3	-1.96 ± 0.11
35	2.6	1.4	0.50 ± 0.03	6.4	1.5	-0.39 ± 0.11	4.2	4.4	-0.79 ± 0.12
40	3.2	2.1	1.67 ± 0.04	3.6	0.8	0.73 ± 0.08	3.8	3.4	0.38 ± 0.06
50	1.7	6.8	4.05 ± 0.14	1.3	5.2	2.93 ± 0.09	2.7	3.1	2.67 ± 0.04

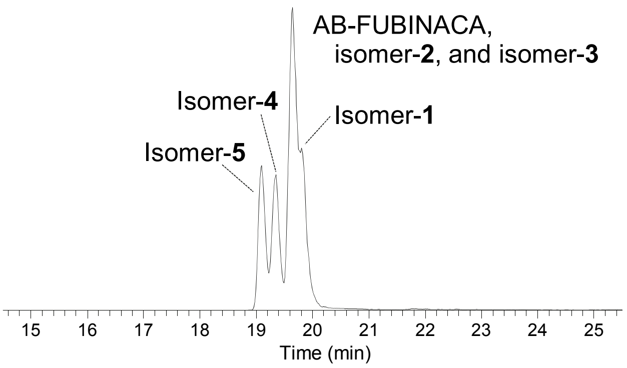

Fig. S1 Extracted ion chromatogram ($[M+H]^+$; *m*/*z* 369) of a mixture of AB-FUBINACA and its five positional isomers using an L-column 2 ODS in gradient mode

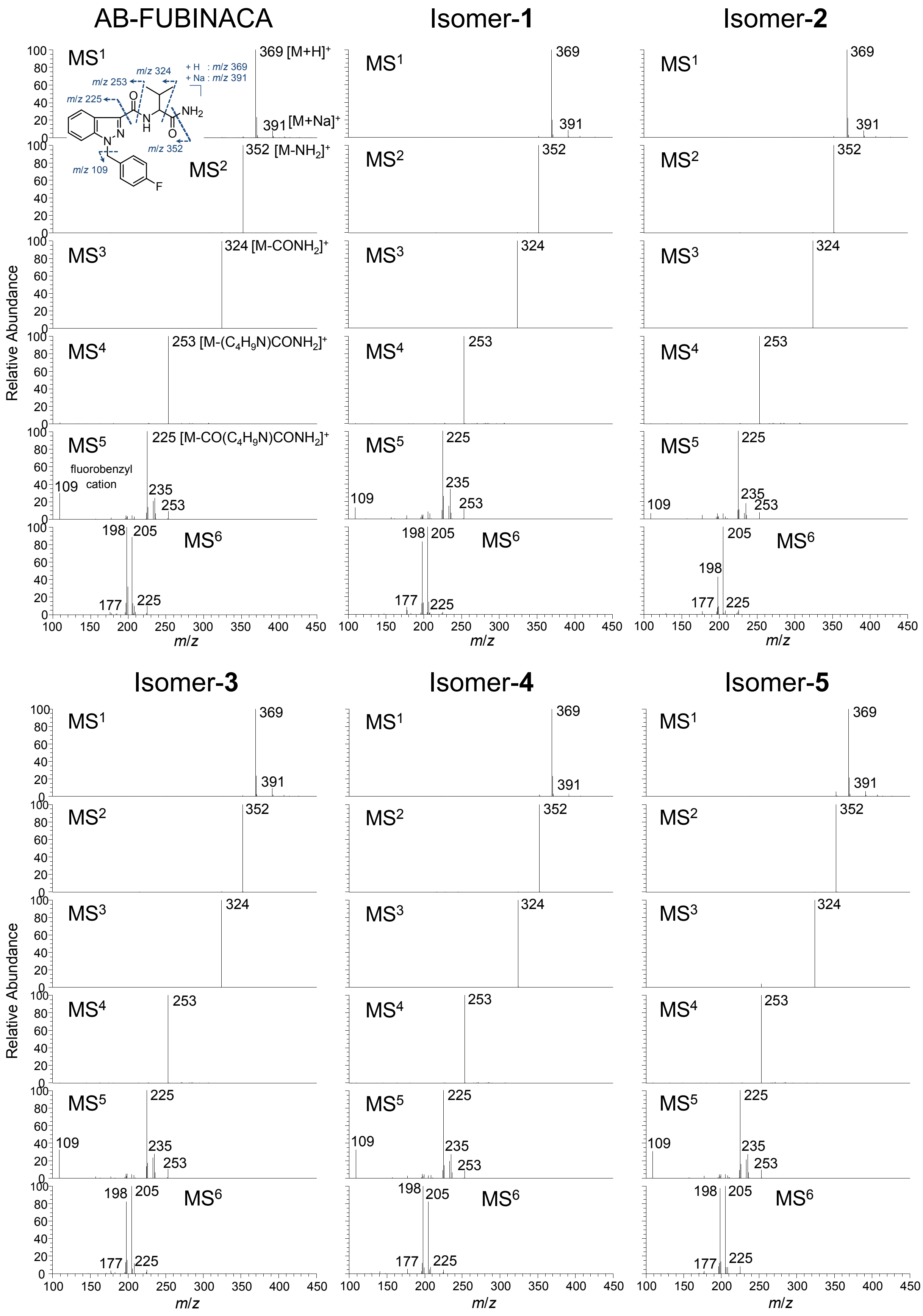

Fig. S2 Electrospray ionization-linear ion trap MS^1–MS^6 spectra of AB-FUBINACA and its five positional isomers in positive mode. The precursor ions at the MS^2–MS^6 stages were set at *m*/*z* 369, 352, 324, 253, and 225, respectively

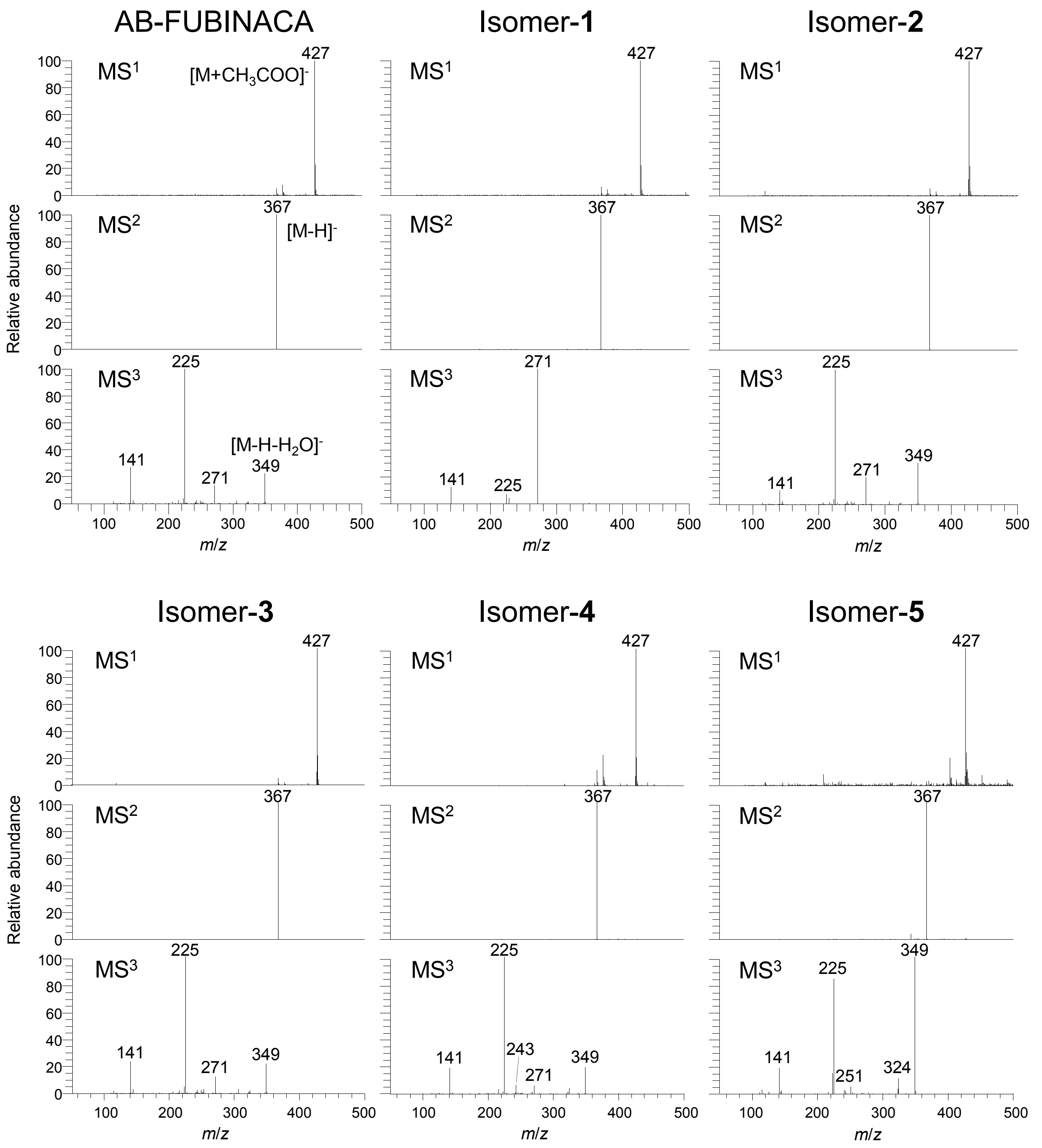

Fig. S3 Electrospray ionization-linear ion trap MS^1–MS^3 spectra of AB-FUBINACA and its five positional isomers in negative mode. The precursor ions at the MS^2 and MS^3 stages were set at *m*/*z* 427 and 367, respectively

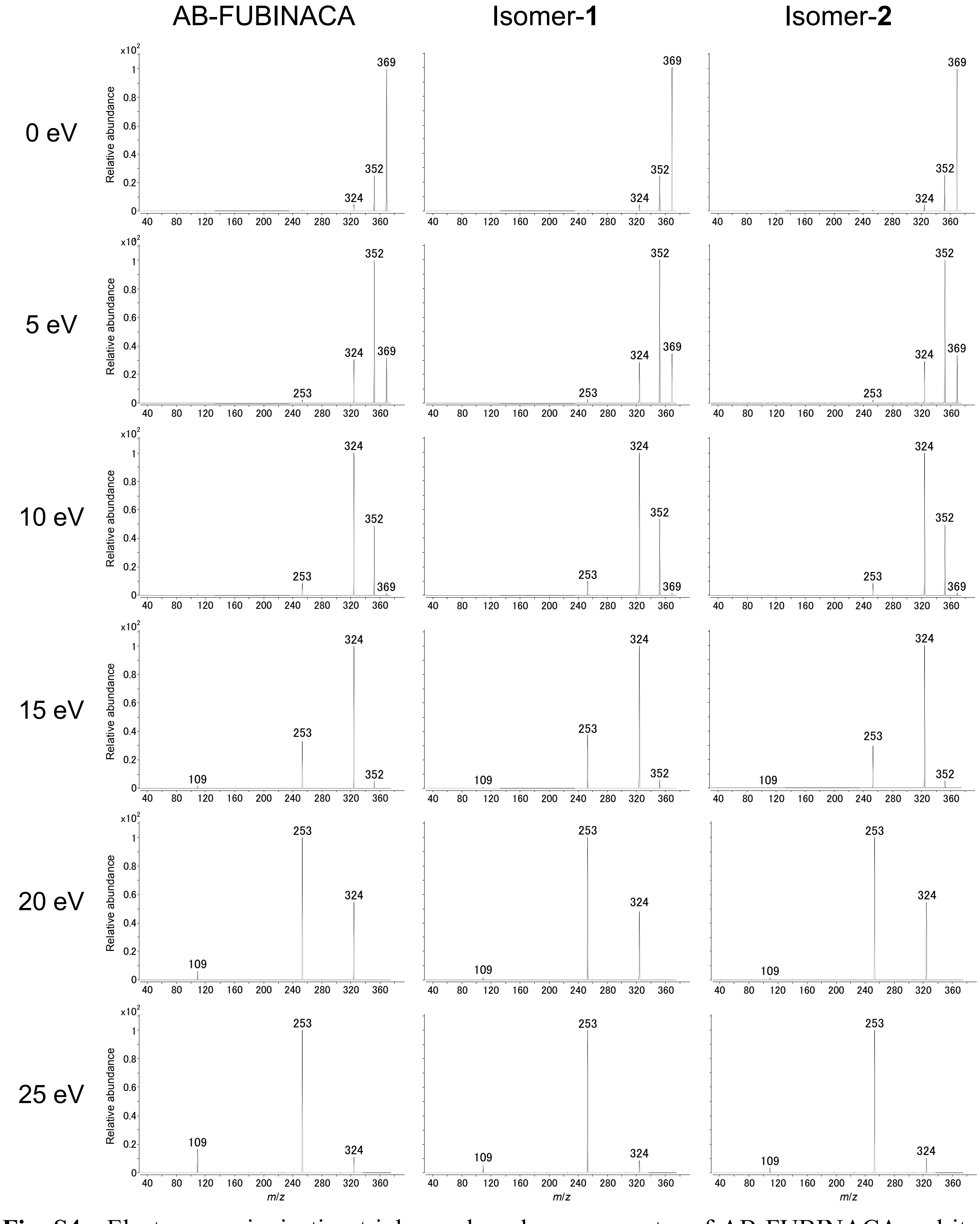

Fig. S4 Electrospray ionization-triple quadrupole mass spectra of AB-FUBINACA and its five positional isomers at collision energies of 0−90 eV for the precursor ions at *m*/*z* 369

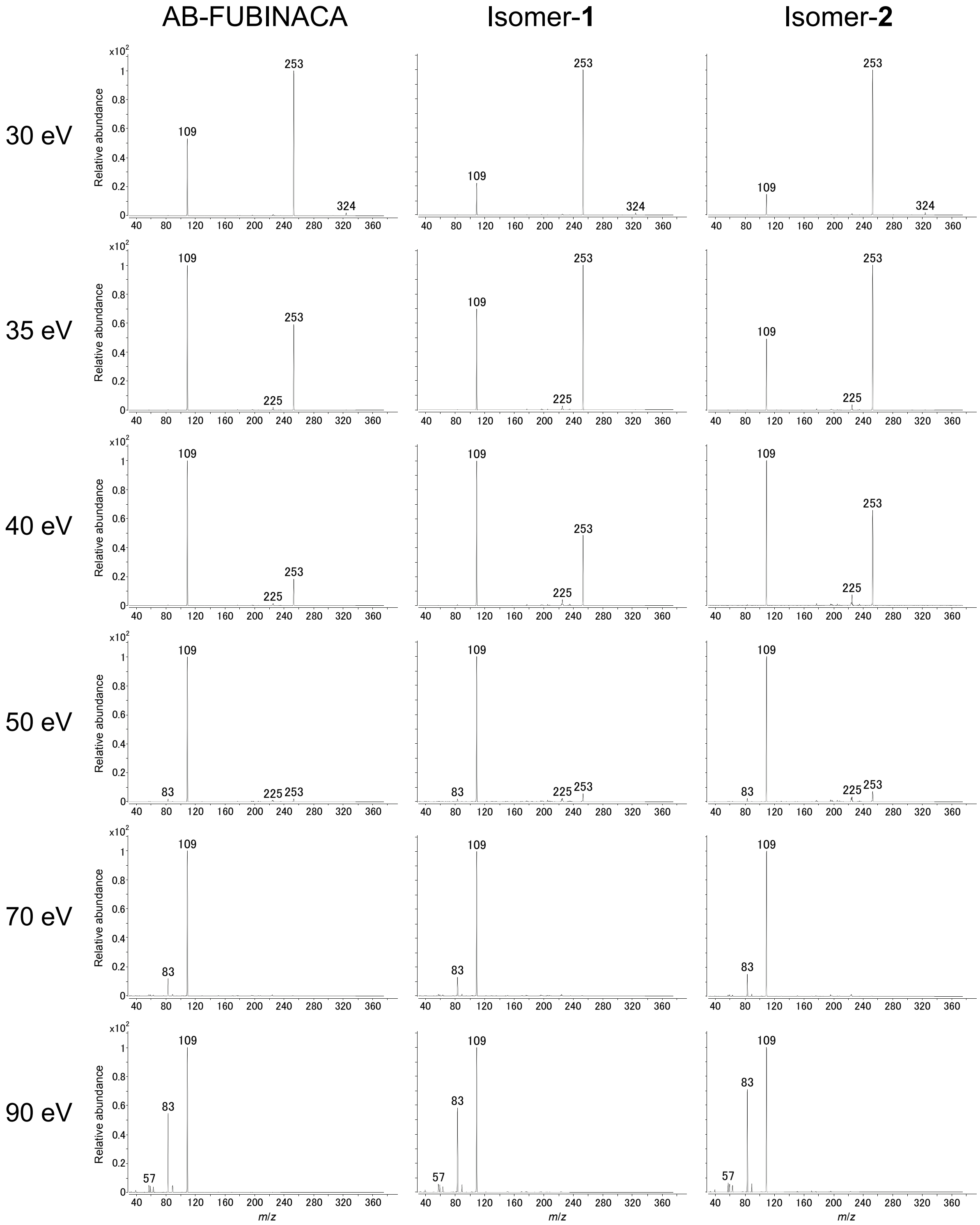

Fig. S4 continued

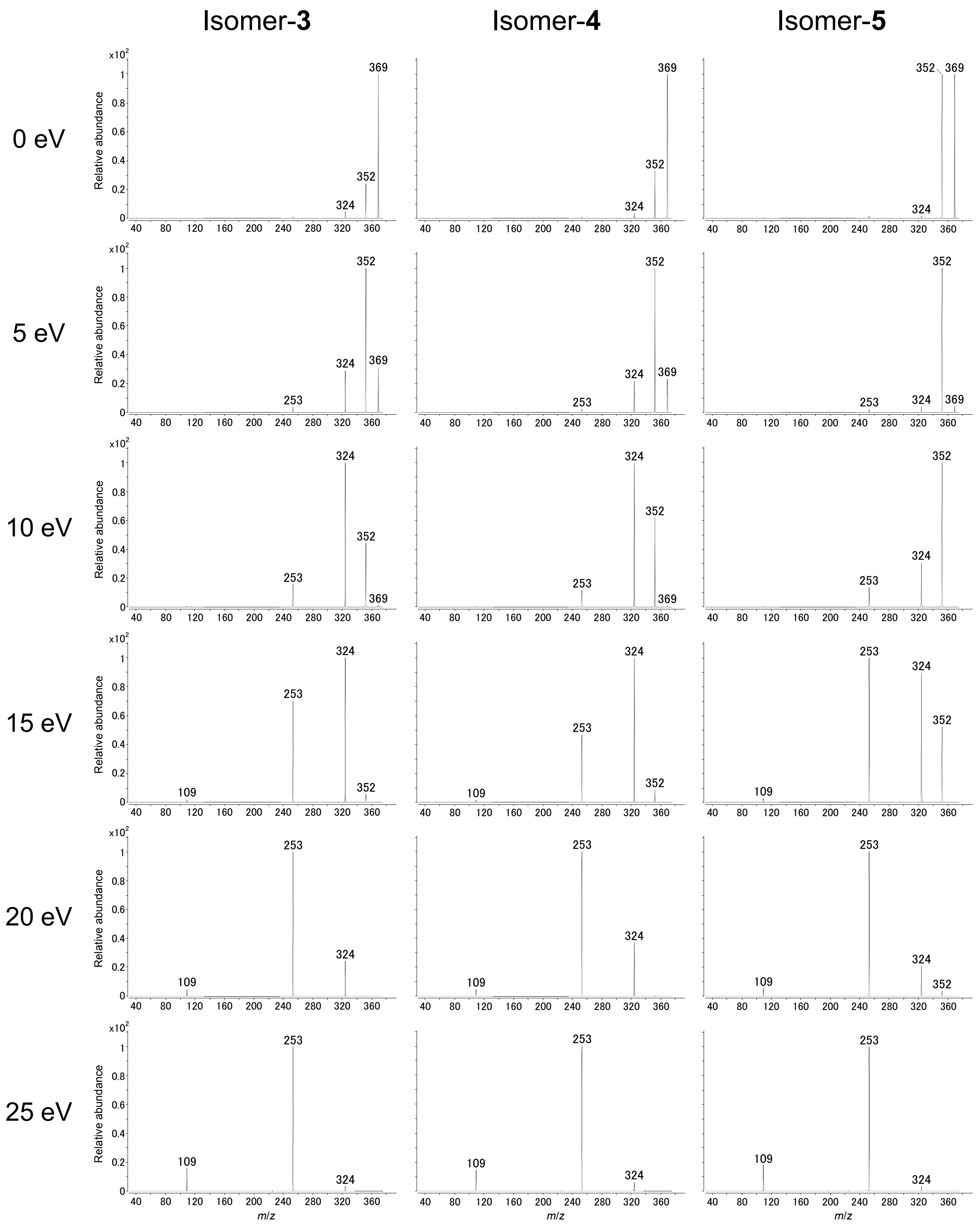

Fig. S4 continued

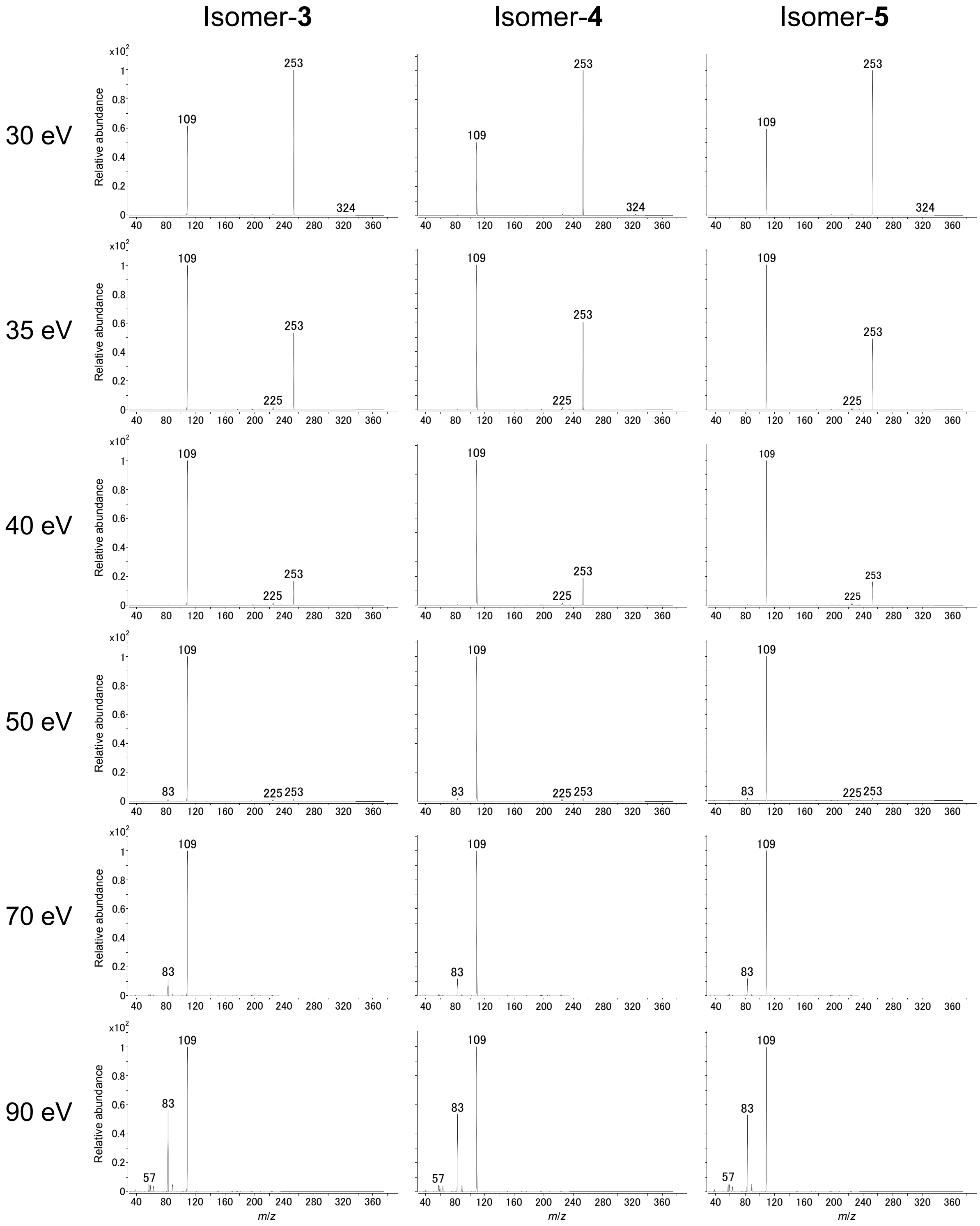

Fig. S4 continued

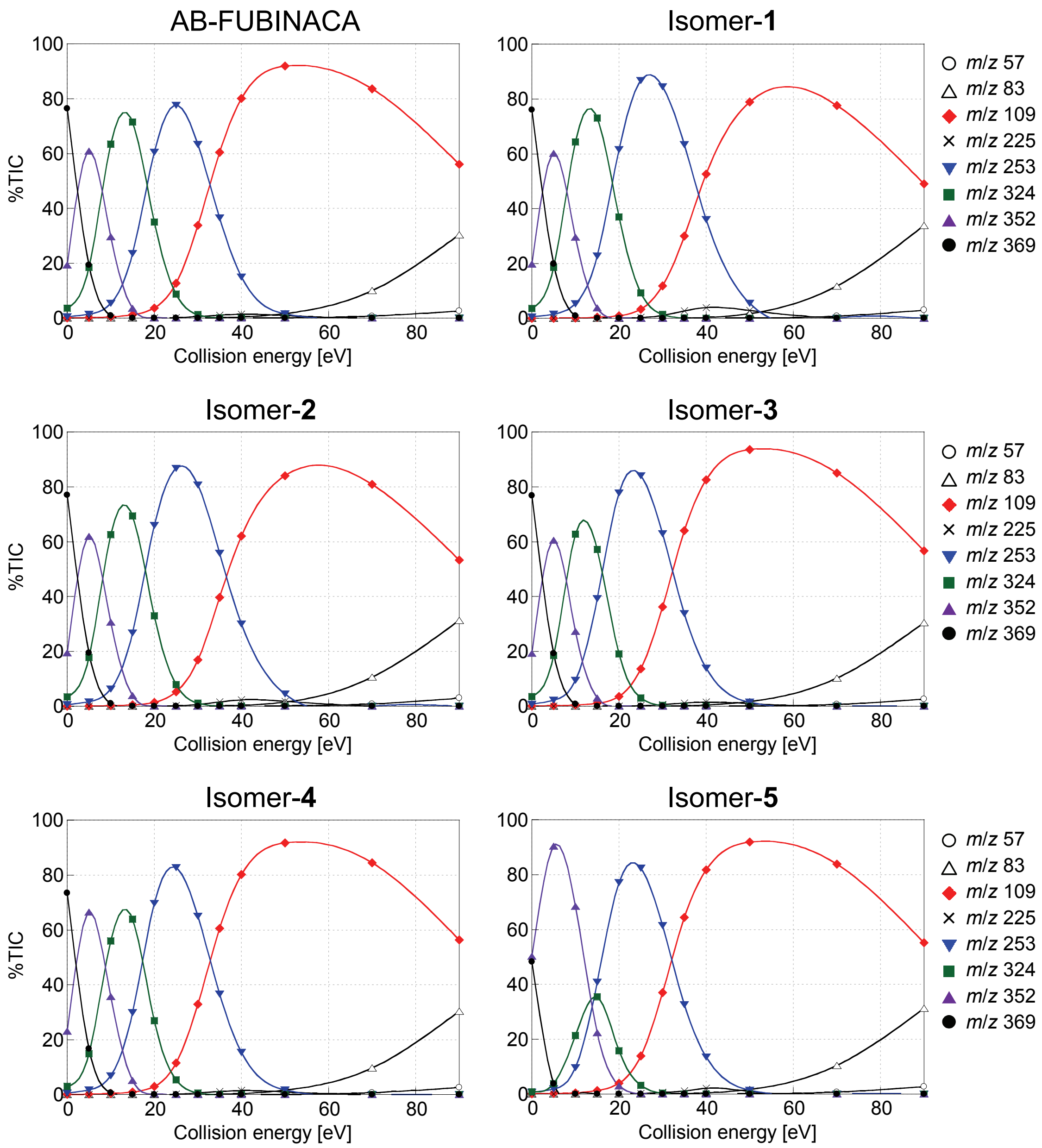

Fig. S5 Breakdown curves of AB-FUBINACA and its five positional isomers for the precursor ion at *m*/*z* 369
